# Large-scale all-atom molecular dynamics alanine-scanning of IAPP octapeptides provides insights into the molecular determinants of amyloidogenicity

**DOI:** 10.1038/s41598-018-38401-w

**Published:** 2019-02-21

**Authors:** Richa Tambi, Gentaro Morimoto, Satoshi Kosuda, Makoto Taiji, Yutaka Kuroda

**Affiliations:** 1 Department of Biotechnology and Life Sciences, Graduate School of Engineering, Tokyo University of Agriculture and Technology, 2-24-16, Nakamachi, Koganei, Tokyo 184-8588 Japan; 2 Computational Biology Research Core, Quantitative Biology Center (QBiC), RIKEN, 6-2-3, Furuedai, Suita, Osaka 565-0874 Japan; 3 Present Address: College of Medicine, Mohammed Bin Rashid University of Medicine and Health Sciences, Dubai, 505055 UAE

## Abstract

In order to investigate the early phase of the amyloid formation by the short amyloidogenic octapeptide sequence (‘NFGAILSS’) derived from IAPP, we carried out a 100ns all-atom molecular dynamics (MD) simulations of systems that contain 27 peptides and over 30,000 water molecules. The large-scale calculations were performed for the wild type sequence and seven alanine-scanned sequences using AMBER 8.0 on RIKEN’s special purpose MD-GRAPE3 supercomputer, using the all-atom point charge force field ff99, which do not favor β-structures. Large peptide clusters (size 18–26 mers) were observed for all simulations, and our calculations indicated that isoleucine at position 5 played important role in the formation of β-rich clusters. In the oligomeric state, the wild type and the S7A sequences had the highest β-structure content (~14%), as calculated by DSSP, in line with experimental observations, whereas I5A and G3A had the highest helical content (~20%). Importantly, the β-structure preferences of wild type IAPP originate from its association into clusters and are not intrinsic to its sequence. Altogether, the results of this first large-scale, multi-peptide all-atom molecular dynamics simulation appear to provide insights into the mechanism of amyloidogenic and non-amyloidogenic oligomers that mainly corroborate previous experimental observations.

## Introduction

Amyloids formed by self-association of misfolded proteins into beta-stranded fibrils are correlated with a number of neurodegenerative diseases^1–5^. A cross β-conformation is a common feature of the structured amyloids^6–8^. The prefibrillar oligomers formed in the early stage of amyloidogenesis are usually transient species making them nearly inaccessible to experimental biophysical techniques^9,10^, and they have been mostly characterized by computational methods^11–13^.

A widely studied disease associated with amyloidogenesis is type II diabetes, which involves Islet amyloid polypeptide (IAPP)^14,15^. IAPP is a 37-residue peptide found in all mammals and co-secreted with insulin by pancreatic β-cells^14^. IAPP amyloid fibers lead to β-cell dysfunction and cell death^16^. Its sequence is highly conserved, but minimal sequence variations observed among species have been reported to affect IAPP’s amyloidogenicity^14,15^. Much experimental and theoretical research works have been carried out to decipher the molecular basis and mechanisms of IAPP amyloids, yet they remain elusive. Human IAPP sequence contains regions such as residues 22–29 (NFGAILSS) that can form amyloids as isolated peptides, similar to those formed by the full-length IAPP^17–20^.

In this work, we aimed at identifying interactions that cause oligomerization and those that cause the formation of β-structures in the oligomeric state at the early stage of amyloid formation by the octapeptide corresponding to residue 22–29 of IAPP. To this end, we performed 100 ns all-atom molecular dynamics (MD) for the wild type and seven alanine-scanned mutants of NFGAILSS. Each MD systems consisted of 27 peptides and approximately 30,000 water molecules and were performed using AMBER 8.0 on a special purpose MD-GRAPE3 computer. To date, this is the largest all-atom MD analysis of the amyloidogenicity of the IAPP octapeptide using large multi-peptide systems containing as many as 27 elongated monomeric peptides in their initial configurations.

## Results

### Cluster and Secondary Structure Analysis

**Figure 1:**
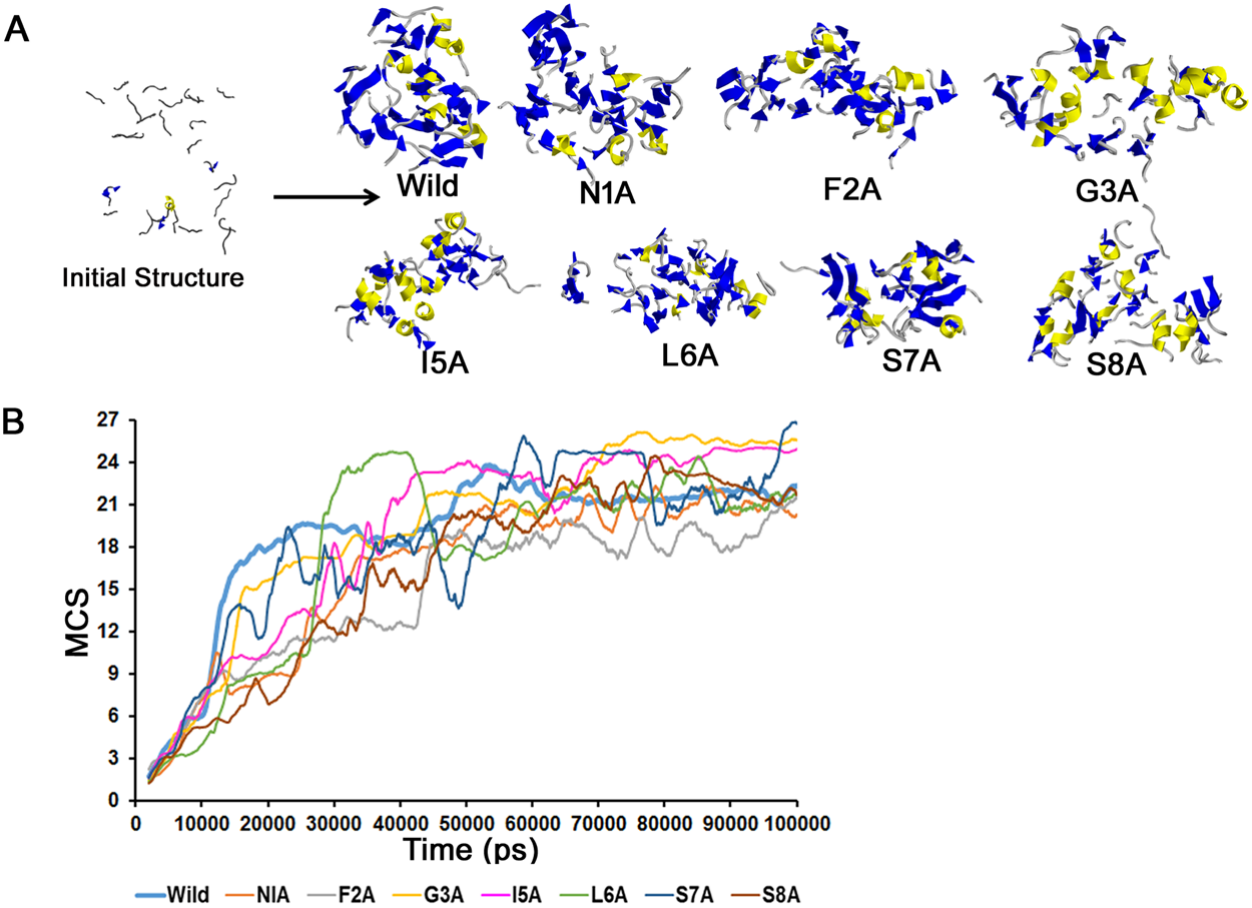
Oligomer formation in wild type and the ala-scanned peptides. (**A**) Snapshot of the initial (1 ns) and later stages (Wild (90.11 ns), N1A (99.30 ns), F2A (79.26 ns), G3A (86.98 ns), I5A (90.63 ns), L6A (88.62 ns), S7A (87.25 ns) and S8A (81.22 ns) peptide clusters- clusters are shown by ribbon models where coil, helices, and sheets are shown in ‘grey’, ‘yellow’ and ‘blue’, respectively) configuration. The ‘initial structure’ shows wild type snapshot at 1 ns which was similar for all the mutants. Initially, 27 peptides were aligned parallelly inside a cubic box [(104 Ǻ)^3^] with approximately 30,000 water molecules which form oligomers during the simulation. These structures were rendered using Rastop (Valadon P., www.geneinfinity.org/rastop/). (**B**) Time dependence of the mean cluster size (MCS). MCS is plotted against time for all the eight systems. Large clusters were observed for all the analogs by the end of the simulation.

Amyloids formation involves the association of monomeric peptide units forming oligomers eventually leading to fibrils, which consist of cross β-sheets^21–23^. Here, we analyzed the oligomer formation through peptide clusters. Both the wild type and the alanine-scanned mutants associated in large clusters (>18 – more than 67% of all peptides in the system – Fig. 1). The wild-type formed clusters containing 18 peptides within the first 20 ns–40 ns, which increased to a 21-peptide cluster by 70 ns, after which it remained constant. The average cluster size during the last 30 ns for N1A, F2A, G3A, I5A, L6A, S7A, and S8A were 20.9, 18.9, 25.5, 24.6, 21.8, 22.8 and 22.4, respectively. Large clusters were thus observed during the 100 ns runs, and we therefore analyzed the secondary structure tendencies of the peptides in order to determine any relationship to their experimentally determined amyloidogenicity.

**Figure 2:**
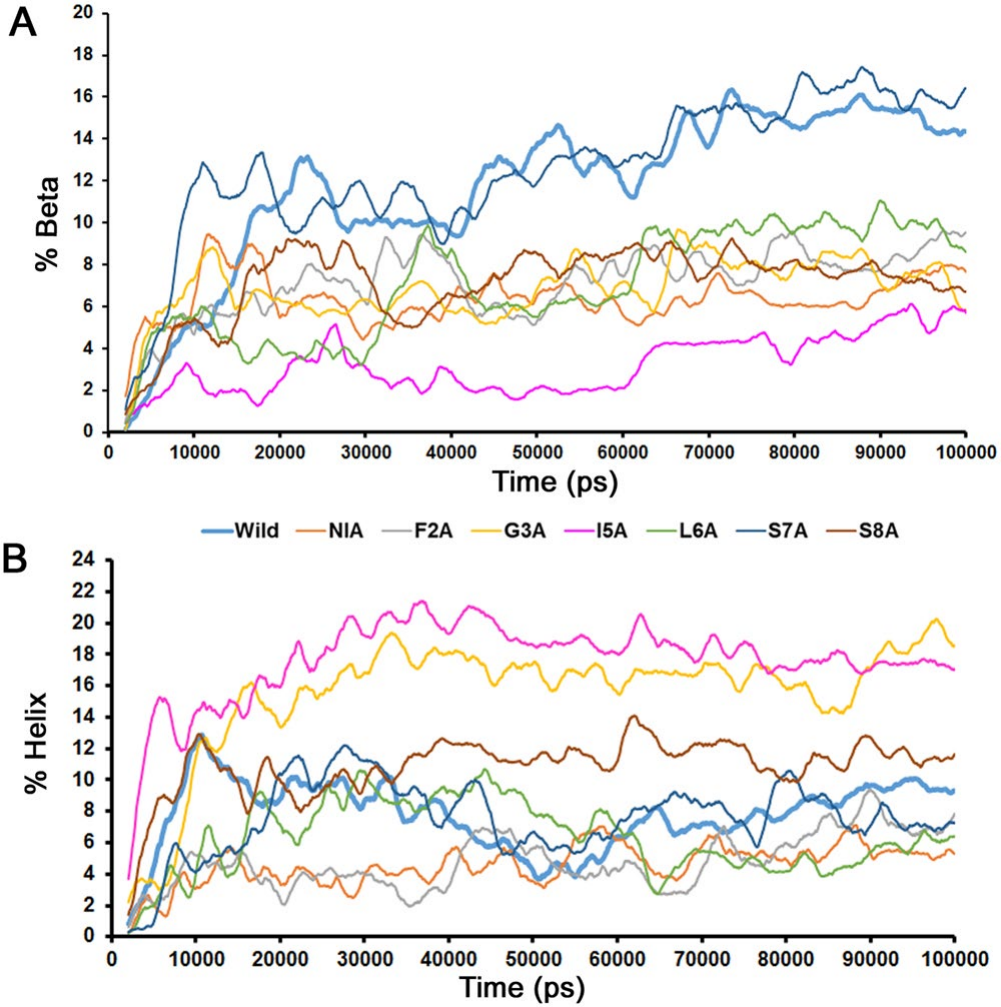
Secondary structural transition observed for all the systems within 100 ns. (**A**) Time dependence of β-structure. Maximum (14–16%) β-structure was observed for Wild-type and S7A peptides and minimum (>6%) was observed for I5A peptide oligomers. (**B**) Time dependence of helical structure formation. Maximum (16–20%) helical content was observed for G3A and I5A, while for all other analogs the content was less than 12%. The percent of helix and β-sheet was calculated using DSSP.

We calculated the structural transition of the peptides, which were initially devoid of any secondary structures to β structures and/or helices within the 100 ns timescale. The wild type and the S7A peptides accumulated up to 14–16% of β structures (Fig. 2A), whereas β structures were 6–10% in all other mutant peptides, and they never exceeded 6% in I5A. Antiparallel β structures were more abundant than parallel ones (Fig. S1). These observations are in line with experiments showing that amyloids formed by the IAPP 20–29 fragment consist of both parallel and anti-parallel β-sheets^24,25^. I5A and G3A accumulated around 16–18% of helical content whereas all other peptides showed lower helical contents (<12% - Fig. 2B). Overall, the content of β structures was higher in the wild type than in any of the alanine-scanned mutants.

### Intrinsic secondary structure preference of monomeric octapeptides

The present calculation starts with 27 isolated peptides, which is very different from previous calculations, where the peptides are preformed into a cross beta-sheet structure^26^. Our calculation is computationally more demanding than previous ones, but it enables an analysis of the amyloid formation from a much wider viewpoint than previous calculations that span only the conformational space around the final cross-beta sheet structure. In particular, we analyzed the intrinsic secondary structure preference of the six monomeric peptides (Wild type & N1A – L6A) by carrying out short 50 ns simulations (Fig. S2). A comparison of the preferred secondary structure of the isolated peptides to that of the oligomerized peptides would enable to determine the effect of peptide association in the formation of beta-structures as discussed thereafter.

The predominant state adopted by all of the peptides was a random coil, but we observed that the wild type and L6A peptides contained more helical residues than beta structures. N1A and I5A favored both helical and beta structures; whereas F2A showed little affinity towards both structures. Helical structures in G3A were observed very early, possibly because two Ala are followed by an Ile, which are helix-favoring residues. Altogether, residues in the monomeric IAPP fragments were mostly in a random coil state, followed by a small fraction of residues in the helical and β -structure states.

### MSM Analysis

**Figure 3:**
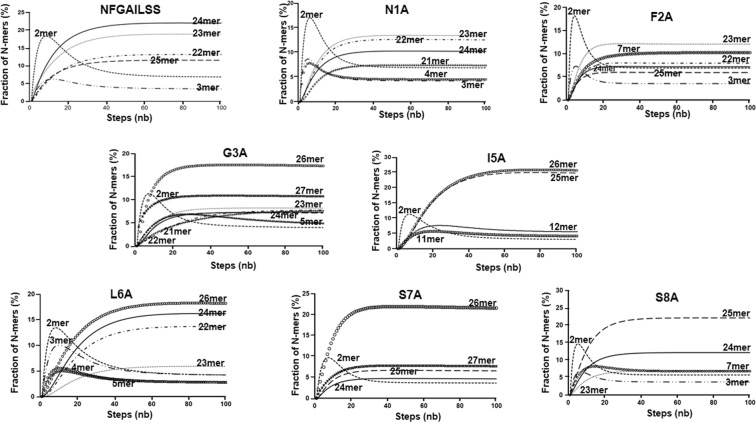
Markov State Model analysis of n-mers fractions. The 100 ns trajectory was used for constructing the transition matrix. Only n-mers fractions that cumulated above 5% are shown for clarity.

Markov state models(MSM) are an efficient method used to extract the distribution of defined macrostates from the molecular dynamics run^27–29^. Here, we used MSM to compute the transition probabilities between the conformations attained by the IAPP fragment. The transition probabilities were calculated such that each propagation step depended only on its previous step and not on any state before that. These probabilities were used to map the distribution of the states. We constructed two types of MSMs: One investigating the distribution of N-mer (dimer, trimer, tetramer …. 27mer) fractions; and a second one where the states were defined by combining the secondary structure and the oligomeric state. The first type of MSM revealed that for almost all the peptides dimeric clusters rapidly accumulated (<10%) before merging into larger clusters (Fig. 3). The dimeric cluster might play the role of a ‘seed’ for forming larger aggregates similar to the amorphous clusters observed in our previous MD calculation using short single amino acids tetrapeptides^28,30–33^. The average sizes of the dominant cluster were 24mer for wild type, 23mer for N1A and F2A, 25mer for S8A and 26mer for the other mutants, reflecting the tendency of all of the peptides to aggregate. In the second MSM, we defined six states as follows: monomeric coil, oligomeric coil, monomeric strand, oligomeric strand, monomeric helix, and oligomeric helix (Fig. S3). A clear transition from monomeric coil to oligomeric coil occurred within 10 ns for all of the systems indicating that the secondary structure preferences of the octapeptide fragments are associated with their oligomerization into clusters rather than their sequences, as we discussed above using MD simulation of monomeric peptides. Table 1 shows the maximum percentages of secondary structures found in the peptides, as calculated from the MSM analysis. Both the wild type and S7A had a higher percentage of β structures (11–12%) than helical structures (7%), and I5A had the highest amount of helical oligomer (16%) and lowest amount of stranded oligomer (3%). Finally, we also observed that in the wild-type, G3A, I5A, and L6A, a small increase (2–4%) of the helical monomeric state occurred and disappeared upon the growth of the oligomeric state (Fig. S3).

**Table 1:** MSM analysis. Percentage of oligomeric helix and oligomeric strand computed from the MSM analysis.

Peptides	Oligomeric helix (%)	Oligomeric strand (%)
Wild	7	11
N1A	5	7
F2A	4	7
G3A	14	6
I5A	16	3
L6A	5	7
S7A	7	12
S8A	11	7

### Inter-peptide H-bonds and side chain contacts

**Figure 4:**
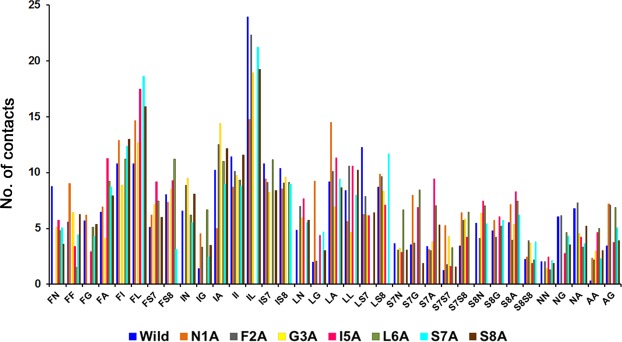
Side-chain contacts analysis. Y-axis represents the absolute number of side chain contacts for thirty-five residue pairs, averaged over the last 30 ns (as explained in the method section and tabulated in Table 3) for all the systems. Ile-leu contacts are evidently the most dominant contacts for wild type and five of its mutants (N1A, F2A, G3A, S7A, and S8A). A maximum number of Phe-Ile and Phe-Leu contacts was computed for I5A and L6A, respectively.

We also examined the contribution of hydrogen bonds and side chain contacts to the formation of clusters. First, the total number of H-bonds as identified by HBPLUS were around 0.3 per residue and similar among all sequences (Table 2). This figure is similar to that of mainchain-mainchain H-bonds observed in the amorphous clustering of tetrapeptides^28^. On the other hand, the number of sidechain-sidechain contacts (see method section) was dependent on the sequence (Fig. 4 and Table 3). Namely, the overall number of contacts between residue pairs was significantly reduced for I5A, F2A, and L6A (Table 3). The largest number of side chain contacts was observed between Ile and Leu in all sequences, but I5A and L6A. The Phe-Leu contacts (10.1%) were dominant in I5A peptide, and a maximum of 6.3% contacts was observed for both Phe-Ile and Phe-Ser8 in L6A. These results combined with the DSSP analysis and MSM plots show that Isoleucine at the fifth position in this octapeptide fragment had the largest influence on β structure formation in the clusters.

**Table 2:** Average number of H-bond per residue between peptides calculated over the last 30 ns using HBPLUS.

Peptide	Total	MM^a^	MS^b^	SS^c^
Wild	0.32	0.19	0.11	0.02
N1A	0.31	0.23	0.07	0.02
F2A	0.33	0.2	0.11	0.02
G3A	0.3	0.14	0.14	0.03
I5A	0.34	0.17	0.14	0.03
L6A	0.4	0.22	0.15	0.03
S7A	0.36	0.22	0.12	0.02
S8A	0.25	0.14	0.1	0.01

^a^‘MM’ denotes H-bonds between main chain-main chain.

^b^‘MS’ means main chain-side chain.

^c^‘SS’ means side chain-side chain.

**Table 3:** Side chain contacts between peptides.

Peptide	Total^d^	HC^e^
Wild	1.12	9.9% (IL)
N1A	0.96	7.1% (IL)
F2A	0.84	12.4%(IL)
G3A	0.91	9.6% (IL)
I5A	0.8	10.1% (FL)
L6A	0.83	6.3% (FS8)^f^ 6.3% (FI)
S7A	0.88	11.1% (IL)
S8A	0.85	10.4% (IL)

^d^Total’ represents the average number of side chain contacts per residue calculated over the last 30 ns.

^e^‘HC’ denotes the percentage contribution of the highest contact over the last 30 ns, and the contacting pair (represented by one-letter code of amino acid residues) is within brackets.

^f^FS8 means a contact between Phe and Ser 8.

## Discussion

Experimental evidence clearly illustrate the context-dependent aspect of the IAPP preferred conformation: It is helical when bound to the membrane, it forms β-sheet in amyloids, and in solution, it is primarily in a random coil conformation^15^. The amyloid forming mechanism of IAPP has been widely studied using sequence fragments, especially the 22–29 residue fragment that has a strong amyloidogenic tendency despite its short sequence.

Here, we used the same fragment and ran a 100 ns simulation with 27 peptides arranged in an ordered manner, and we have previously shown that the simulation results are independent of the initial peptide’s configuration^28^. To date, the present calculation starting with isolated peptides is conceptually different from previous calculations starting with peptides in a cross β-sheet structure^26^, where the analysis is restricted to the conformational space around the cross β-sheet structure. Moreover, the all-atom point charge force field ff99 used here do not favor β structures^34^, and the individual peptides had a marked preference for helices (besides random coil). Yet, we observed a considerable amount of β-structures in the wild type as well as some of the mutant peptides indicating that this preference for β structures originates from the association of peptides into clusters.

The formation of amyloid structures in IAPP derived peptide fragments has been intensively investigated. For the purpose of the discussion, it is important to note that discrepancies exists among the experimental results, which might originate from sequence but also some experimental conditions and sample handling. Specifically, two research groups^26,35^ scrutinized the amyloid-forming tendency of IAPP fragments and its ala-scanned mutants using various experimental techniques. Azriel and Gazit^35^ used residue 22–29 (NFGAILSS), which is the sequence that we used in our calculations, and observed higher amyloid aggregation in wild type, N1A and G3A, which was followed by I5A and L6A and a complete loss of amyloidogenic aggregation was observed in F2A, using congo red staining experiments. On the other hand, for hexapeptide fragments (residue 22–27 NFGAIL^26^), a characteristic birefringence indicating the presence of amyloid fibrils was observed only for the wild type sequence. Moreover, the electron microscopy experiments reported fibrils for all octapeptides (residues 22–29) but F2A^35^, whereas in Zanuy *et al*.^26^ experiments, only the wild type hexapeptide (NFGAIL) had typical fibril morphology. In this study, the wild type and S7A had the highest β-structural content, and the lowest was observed for I5A. This is in line with the octapeptide experiments^26,35^, which showed that the wild-type was highly amyloidogenic whereas the amyloidogenic tendencies of the mutant peptides were considerably reduced. S7A and S8A peptides were not examined in their experiments so that no direct comparison with our calculation is possible.

Our calculations indicated that the largest number of side-chain contacts for the wild type octapeptides and all its analogs except I5A and L6A was between Ile and leu. In L6A, the largest number of contacts were found between Phe and Ile, and the least amount of β-structure was observed in I5A, which suggested that isoleucine at the fifth position in this octapeptide plays an important role in the formation of β-structures. This observation is corroborated by the fact that many of homologous IAPPs have an isoleucine at this position, and most of these IAPPs are amyloidogenic^15^. Furthermore, it was also shown using a thioflavin-T assay that the I26P (corresponding to I5P in our residue numbering) mutation inhibited fibrillation^36^.

In contrast to previous studies where phenylalanine stacking was identified as a determining factor for fibril formation^26,35^, we did not observe any significant number of stacked phenylalanines, Stacked phenylalanines was readily reproduced in our previous all-atom MD simulation using tetra-phenylalanine peptides^28^, and it is thus reasonable to argue that the lack of phenylalanine stacking is not an artifact associated to the force field or to insufficient sampling. Nevertheless, our calculations deal only with the very early stage of oligomerization and, longer simulation times with replica exchange might reveal a contribution for the stacking of phenylalanines^37,38^. Finally, let us note that some experiments suggested that IAPP amyloidogenesis was caused by the side-chain hydrophobicity and β-sheet propensity rather than aromatic stacking interactions^39,40^, which would be in line with our results. Recently Bakou *et al*.^41^, have shown that both Phe 23 and Ile 26 (2^nd^ and 5^th^ position in this fragment) in IAPP are important for self-assembly of amyloids, again in line with our results. Overall, we observed that hydrophobic interactions are an essential factor for the association of IAPP peptides into β-rich clusters.

## Conclusion

This study is part of a series of experimental^30–32^ and theoretical works^28,33^ conducted for understanding and controlling protein and peptide aggregation and solubility^42^. In particular, this study significantly extends our previous all-atom MD simulations of systems containing up to 54 tetra-peptides, which provided insights into the formation of amorphous aggregates^28^. Here, we examined the clustering of amyloidogenic IAPP octapeptide and its ala-scanned mutants, using multi-peptide systems in the absence of preformed beta-sheet structures, which to the best of knowledge is the first such attempt. Our calculation starting with isolated peptides is conceptually different from previous calculations starting with pre-formed β-sheets. The results are expected to provide a model for understanding the mechanism of amyloid formation in a short, amyloid-prone peptide, rather than characterising the stability and dynamics of amyloid structures formed by IAPP peptide fragments. The significant finding was that all peptides formed large clusters with a similar number of H-bonds, as observed in our previous MD simulation of tetrapeptides^28^, but the content of β-structures was strongly peptide-dependent and the fractions of β-structures were roughly in line with experimental results. The wild type and S7A mutant had the highest content of β-structures and I5A the lowest one. Inter-peptide side chain contacts in the wild type sequences were most abundant between Ile and Leu, indicating the significance of hydrophobic interaction for driving the association process into the beta-rich clusters. We believe that large-scale simulations with increased computational power, such as this study, will provide an even deeper understanding of the mechanism of the oligomerization of amyloidogenic and non-amyloidogenic peptides.

## Material and Methods

### Model Systems

We performed MD simulation for NFGAILSS (wild type) and its seven alanine-scanned mutants denoted by N1A, F2A, G3A, I5A, L6A, S7A, and S8A. All the structures were constructed using the LEaP module of Amber 8.0^43^. The peptides were capped at both the N- and C- terminal by N-methyl (-NHCH3) group and an acetyl (CH3CO-) group, respectively, in order to eliminate the terminal charge effect. The initial configuration consisted of 27 isolated peptides aligned in a parallel orientation, on a 25 Å grid. The peptides were immersed in a cube of 104^3^Å^3^ containing approximately 30,000 water molecules, which corresponded to a peptide concentration of ~40 mM.

### MD Simulation

All calculations were performed on a RIKEN’s special purpose supercomputer MDGRAPE-3^44,45^. For each peptide, two sets of MDs were performed simultaneously: 100 ns MD runs with 27 elongated peptides positioned in a parallel orientation in a 104^3^Å^3^ box, and 50 ns runs of single peptides. The MD simulation was carried out using the Amber 8.0 software package, using the all-atom point charge force field ff99^46^ and TIP3P water model^47^. SHAKE algorithm was used to constrain the bond length and a 2 fs time step was used in all the simulations. Particle mesh Ewald (PME) method was used for the long-range electrostatic interactions and a radius cut-off of 14 Å was used. Each system was subjected to energy minimization using the steepest descent protocol followed by a conjugate gradient procedure. After 5000 steps of energy minimization, the systems were gradually heated from 0 K to 300 K at a heating rate of 6 K/ps. Thereafter, a constant temperature and pressure of 300 K and 1 atm, respectively, was maintained with a coupling constant of 1 ps. Trajectories were saved every 10 ps for further analysis. Total simulation time for each 100 ns run was approximately 11 months and each simulation generated around 70 Gbyte of data.

### Cluster and Secondary Structure Analysis

Peptide aggregation was assumed when peptides formed ‘clusters’^28^. A cluster was defined when two or more peptides were in contact, i.e., when the distance between any two atoms of two different peptides was less than the sum of their Van der Walls radius. We defined a cluster size (*CS*) as the number of peptides constituting a cluster, and we calculated the mean cluster size (*MCS*) as

1 $$MCS=\sum _{i=1}^{N}C{S}_{i,t}/N$$

where *CS*_*i*,*t*_ denotes the cluster size to which peptide *i* belongs at time *t*, and *N* is the total number of peptides in the system (27 here).

The secondary structures were identified using DSSP^48^. β-structures were defined by beta bridges (denoted as ‘B’ in DSSP) and extended beta strands (denoted as ‘E’). Helices were the sum of 3_10_ (denoted as ‘G’), α (denoted as ‘H’) and π (denoted as ‘I’) helices.

### MSM Analysis

We used a Markov state model analysis for extracting two types of information from the MD simulations^27–29^. First, we constructed MSM describing the transition between the 27 cluster size states (monomer, dimer, trimer …. twenty-seven mer). Second, we constructed an MSM describing the residue-level transition between 6 states: monomeric coil, oligomeric coil, monomeric strand, oligomeric strand, monomeric helix, and oligomeric helix. We calculated the number of occurrence of each state (*N*) and constructed the transition matrix *T* (*S*_*i*_, *S*_*j*_) describing the transition among the states. We used the complete 100 ns data for constructing a 27 × 27 transition matrix for the first type of MSM and a 6 × 6 transition matrix for the second type of MSM. Transition matrix calculates the total number of transition from state *i* to state *j*:

2 $$T({S}_{i},{S}_{j})=\sum _{t=0}({S}_{i,t}\to {S}_{j,t+1})$$

where *S*_*i*,*t*_ and *S*_*j*,*t+1*_ represent states *i* and *j* at time *t* and *t* + *1*, respectively. Next, we calculated the transition probability *P*:

3 $$P({S}_{i},{S}_{j})=T({S}_{i},{S}_{j})/{N}_{i}$$

where *N*_*i*_ is the total number of occurrences of state *i*. Finally, the fraction of trajectories in each state after *n*-propagation step was computed as the row vector *π(n)*.

4 $$\pi (n)=\pi (0)\times {P}^{n}$$

where *π*(0) is a row vector containing the initial fractional populations.

### H-Bond and Side chain contacts

The number of hydrogen bond interactions was calculated using HBPLUS^49^. The side chains of two residues belonging to two different peptides were considered to be in contact when any of the side chain aliphatic carbon atoms were within 5.5 Å. Inter-peptide phenylalanine residues were assumed to be interacting when the distance between the geometrical center of the aromatic ring was within 5 Å^26,28^.

## Supplementary information

supplemental data
