# Supplementary material for: Large-scale all-atom molecular dynamics alanine-scanning of IAPP octapeptides provides insights into the molecular determinants of amyloidogenicity
Source: Sci Rep. 2019 Feb 21;9:2530. doi: 10.1038/s41598-018-38401-w (PMC6384915; doi:10.1038/s41598-018-38401-w)

Supplementary Information

**Large-scale all-atom molecular dynamics alanine-scanning of IAPP octapeptides provides insights into the molecular determinants of amyloidogenicity**

**Richa Tambi**1,#**, Satoshi Kosuda**1**, Gentaro Morimoto**2**, Makoto Taiji**2**, Yutaka Kuroda**1,*

1Department of Biotechnology and Life Sciences, Graduate School of Engineering, Tokyo University of Agriculture and Technology, Nakamachi, Koganei, Tokyo 184-8588, Japan

2Computational Biology Research Core, Quantitative Biology Center (QBiC), RIKEN, 6-2-3, Furuedai, Suita, Osaka 565-0874 Japan

#Present Address: College of Medicine, Mohammed Bin Rashid University of Medicine and Health Sciences, Dubai, 505055, UAE

* Corresponding author Email: [ykuroda@cc.tuat.ac.jp](mailto:ykuroda@cc.tuat.ac.jp); Phone/Fax: +81-42-388-7794

Figure S1: Distribution of (a) anti-parallel and (b) parallel β-structures. DSSP calculations indicated the presence of both parallel and antiparallel β structures for all the peptides.


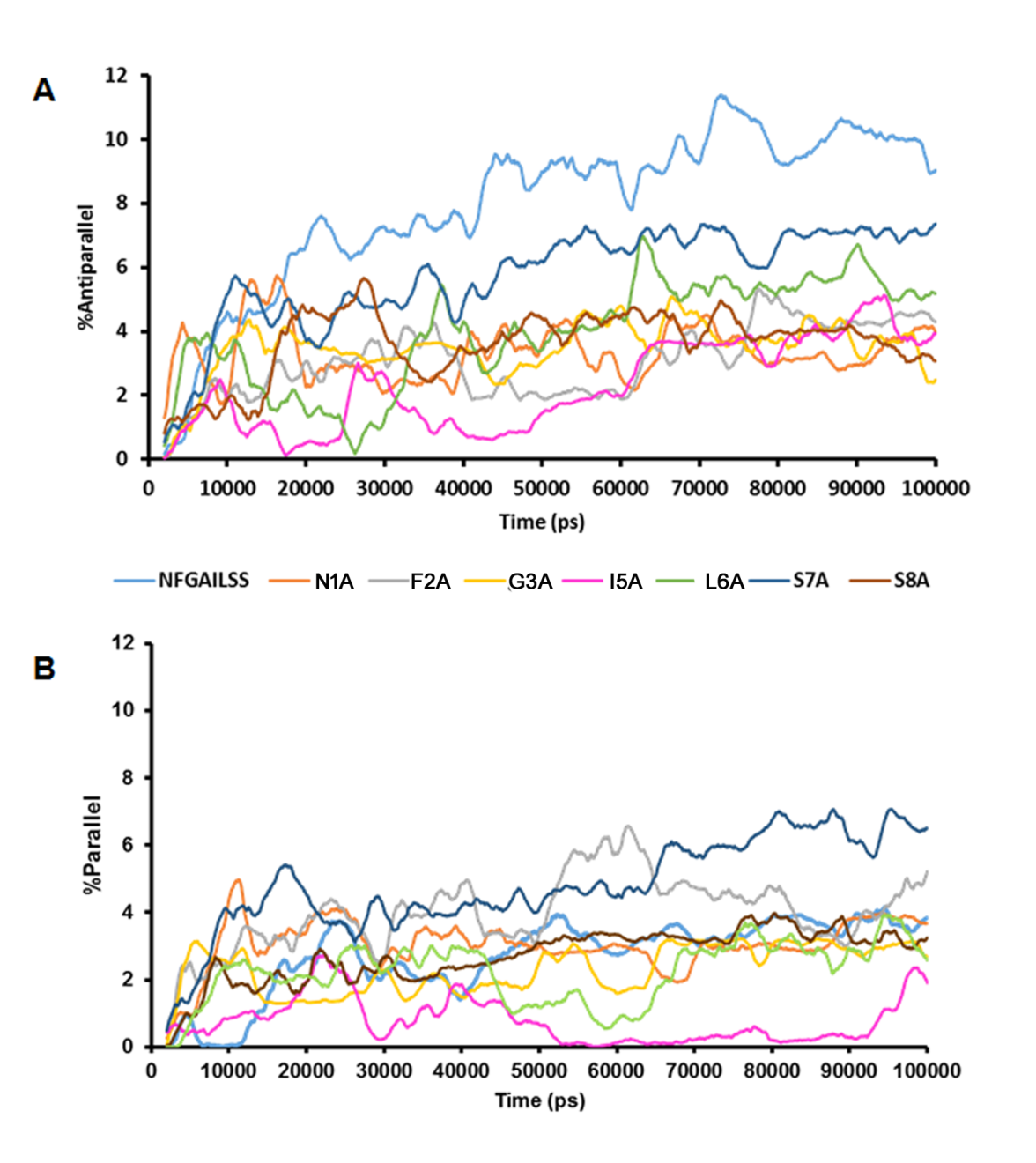


Figure S2: Single peptide simulation results. Percentage of coil shown only for wild type. The intrinsic secondary structure preferences of the six monomeric peptides (Wild type & N1A – L6A) were analyzed by carrying out short 50ns simulations.


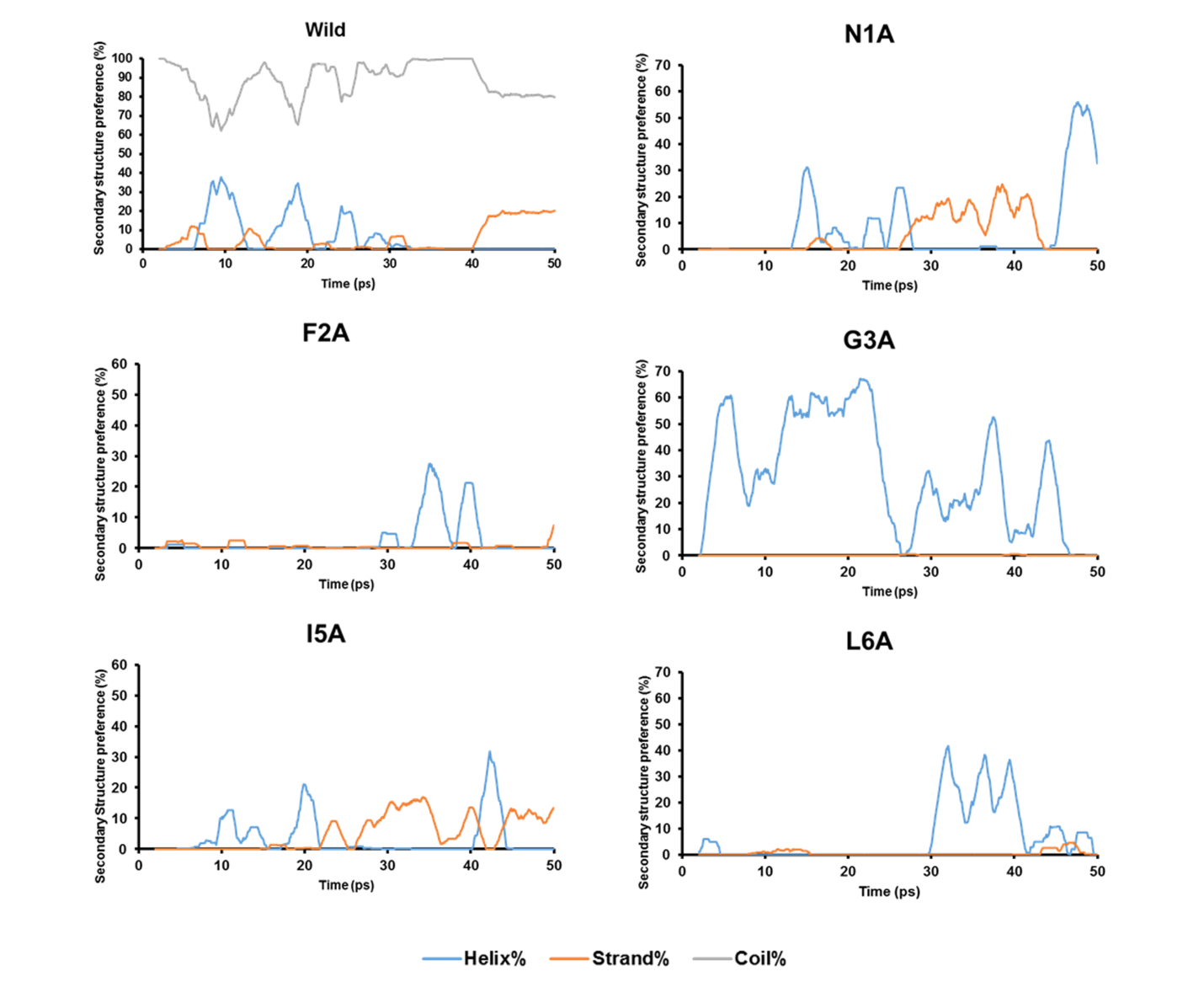


Figure S3: 6 state MSM. Markov state model analysis describing the residue-level transition between 6 states: monomeric coil, oligomeric coil, monomeric strand, oligomeric strand, monomeric helix, and oligomeric helix


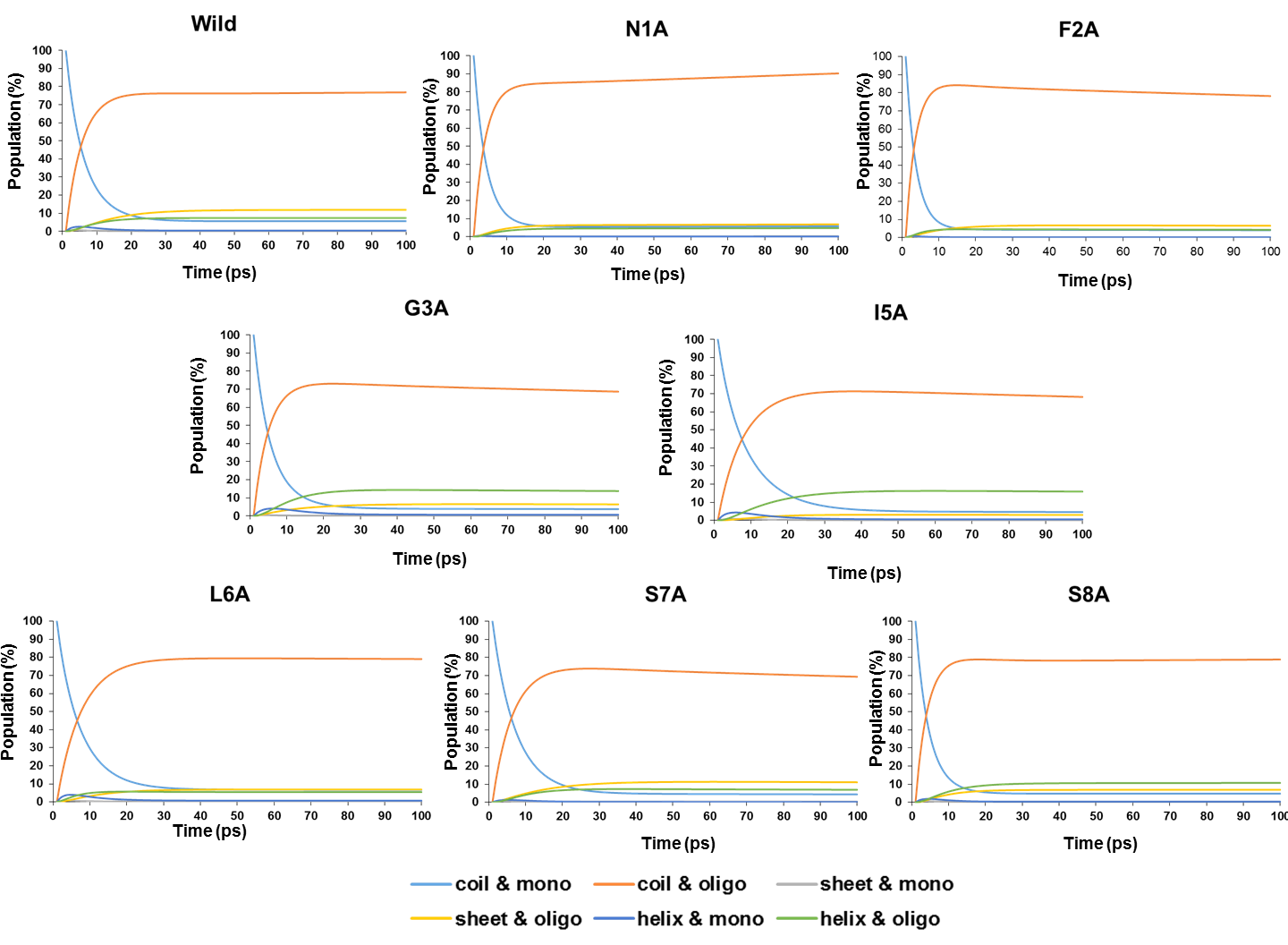

Supplement: Supplementary file 1 — supplemental data [file 41598_2018_38401_MOESM1_ESM.docx]
